# Applying authoritative knowledge to better understand preparation for breastfeeding

**DOI:** 10.3389/fgwh.2025.1540376

**Published:** 2025-03-31

**Authors:** Margaret S. Butler, Sera L. Young, Lauren Keenan-Devlin

**Affiliations:** ^1^ Department of Anthropology, Northwestern University, Evanston, IL, United States; ^2^ Center of Excellence in Maternal and Child Health, School of Public Health, University of Illinois Chicago, Chicago, IL, United States; ^3^ Institute for Policy Research, Northwestern University, Evanston, IL, United States; ^4^ Department of Obstetrics and Gynecology, Endeavor Health, Evanston, IL, United States; ^5^ Department of Obstetrics and Gynecology, University of Chicago Pritzker School of Medicine, Chicago, IL, United States

**Keywords:** authoritative knowledge, breastfeeding, infant feeding, lactation, prenatal information, biomedical, social network, lived experience

## Abstract

**Introduction:**

In this qualitative study, we employ the construct of authoritative knowledge to better understand how birthing people prepare for breastfeeding experiences postpartum. This construct has seldom been applied to the postpartum period, despite its application by reproductive anthropologists to pregnancy and childbirth experiences cross-culturally. Consistent with these applications, we define authoritative knowledge domains by the purveyors. We aimed to characterize the acquisition and valuation of information sources participants used to prepare for breastfeeding.

**Methods:**

Twenty-five participants were recruited from a hospital-based pregnancy study in Chicagoland, Illinois, USA to complete interviews between November 2020 and March 2021. Audio recorded interviews were coded using *a priori* themes and iterative code development. Codes were used to characterize information sources and the designation of three domains of authoritative knowledge: biomedical, social network, and lived experience.

**Results:**

All participants received information about breastfeeding from both biomedical and social network domains, with those with prior child rearing experiences also using the personal experience domain. Use of online resources like pregnancy tracking apps and social media platforms resulted in the domains of authoritative knowledge overlapping. Participants valued information from health care providers the most but found social network information was more accessible and fulfilled their desire for experiential information.

**Discussion:**

In this first application of authoritative knowledge within the context of infant feeding, participants consistently cited biomedical sources as the most accurate and important. However, they cited barriers to gaining this information such as the short duration of prenatal appointments and the challenge of completing prenatal education courses. Many participants sought evidence-based information about breastfeeding on apps, social media, and websites, however content and quality across platforms varies significantly. This may be an avenue to improve access to reliable and helpful breastfeeding information.

## 1 Introduction

Human infant feeding has required substantial social support throughout evolutionary history, evidenced by widespread use of cooperative breeding and alloparenting strategies across time and cultures (1, 2). Historically and contemporarily, birthing people have acquired information about parturition and child rearing from mothers, grandmothers, female kin, and friends (3), as well as from professionals within institutions and organizations as they gained prominence (4–6). In the age of the internet, social networks have vastly expanded, as has access to seemingly endless variety of information and resources for birthing people to prepare for pregnancy, birth, and the postpartum period (7, 8).

Expecting and new parents actively seek out infant feeding information (9, 10) and increasingly do so online (11). Parents in the United States (US) report significant pressure when deciding how to feed their infant, experiencing tensions between personal and work responsibilities, available resources, sociocultural norms and expectations, and public health recommendations to breastfeed (12), i.e., to feed human milk to their infant, for at least 6 months exclusively and 12–24 months with supplemental solids (13, 14). Importantly, the decision to breastfeed is not a neutral choice devoid of influence by sociocultural, economic, and political factors.

Most parents decide to breastfeed, reflected in US breastfeeding initiation rates of 84%, but less than half of those who intend to breastfeed actually meet their goals for exclusive breastfeeding or duration (15). Only 55.8% of US infants are breastfeeding at 6 months, less than half of those infants are receiving exclusively breastmilk as recommended, and just 35.9% are still breastfeeding at 12 months (16). Globally, there are similar trends in breastfeeding rates, 95% of infants have ever breastfed, however 44% of infants are exclusively breastfed up to 6 months (17, 18) with the World Health Organization European Region reporting the lowest rate of exclusive breastfeeding at 6 months at approximately 25% (19). Rates of continued breastfeeding vary substantially by country and region, for example 23% of infants in the Middle East and North Africa, compared to 70% of infants in South Asia (17).

The top 3 barriers to breastfeeding initiation and maintenance that parents report include lack of functional lactation support after discharge from the hospital; perceived insufficiency of milk supply; and separation from the infant in the first few weeks after birth to return to work (15, 20, 21). Across nations, there are broader sociopolitical and economic (e.g., policies for paid leave; workplace protections; marketing of commercial milk formulas) as well as cultural barriers (e.g., prelacteal feeds) hindering families’ ability to meet breastfeeding recommendations (18, 21, 22). Overcoming these barriers relies in part on access to knowledge about lactation during pregnancy to prepare for the realities of breastfeeding, human milk feeding, and milk management, as well as access to information advice, and support when issues arise (12, 23, 24). Despite the primacy of this knowledge in shaping breastfeeding trajectories, there is scant information on knowledge sources utilized by and valued by birthing people in the US, which could inform healthcare and public health strategies for improving breastfeeding outcomes. This study aims to address this gap.

### 1.1 Theoretical framework: authoritative knowledge

Here we apply the construct of authoritative knowledge to understand how families prepare for and navigate infant feeding, specifically breastfeeding. Developed in the field of reproductive anthropology, authoritative knowledge refers to the knowledge system(s) which dominate in a particular context, and is/are used to guide decisions and actions (25, 26). Simply put, authoritative knowledge can be understood as the “right” knowledge that an individual needs to navigate a particular system or power structure. The construct of authoritative knowledge has predominantly been used to understand how individuals make meaning of their labor and birth experiences (25–28). Authoritative knowledge is persuasive because it feels rational, people accept it because it is validated and reinforced, but because they are actively engaged in its production and reproduction, whether consciously or not. For example, Liamputtong and Kitisriworapan (26) illustrated that rural Thai women utilized biomedical knowledge to confirm pregnancy with a home test before engaging prenatal medical care, and they also relied on traditional knowledge conveyed by older female kin for postpartum healing, including restrictions on diet and activity due to the inaccessibility of biomedical care postpartum. As this example demonstrates, authoritative knowledge systems are defined by who “owns” and/or purveys the information (doctors, midwives, and older female kin). Also exemplified above is that multiple domains of authoritative knowledge often co-exist and interact to shape individuals' understanding and experience (29).

In the US, biomedicine is the dominant knowledge system for health and healthcare, reified by the centralization of pregnancy and infant care in the medical system. Ninety-eight percent of deliveries in the US occur in hospitals (30) and more than 92% of infants receive pediatric care in their first year of life (31). Authoritative knowledge can help to identify how both social systems and power dynamics shape lived experiences like pregnancy and/or birth outcomes (58). Anthropologists have demonstrated that pregnant Americans value biomedical perspectives on pregnancy and birth in large part because they are normalized as the standard during pregnancy and childbirth (25, 27, 58, 59). Simultaneously, indigenous, folk, and even embodied knowledge systems derived from cultural practices and collective experiences continue to inform practices related to pregnancy, birth, and postpartum for various cultural and ethnic groups within the US and globally (26, 28). For example, one qualitative study demonstrated how within the context of pregnancy, American birthing people make genetics meaningful as it relates to their future children's heritability via the mediation of different types of knowledges, including intuitive, embodied, expert, sociocultural, and authoritative knowledges (32). Hurst and colleagues explained how participants understood genetics to be strongly influenced by family, culture, and their own internal and external environments of their pregnancies—meaning they accepted the authoritative knowledge of prenatal testing but filtered this information through these other lenses to make their experiences more meaningful (32). Of note, we found only one example of this construct being applied to understand postpartum experiences (33), and our study goes beyond the theoretical application of authoritative knowledge by explicitly measuring the sources of information participants accessed to learn about and prepare for infant feeding.

### 1.2 Objectives

This study aimed to (1) identify sources and types of authoritative knowledge that pregnant individuals acquired in preparation for infant feeding and (2) characterize the degree to which individuals ascribed value to those information sources. Across these objectives we examined differences in acquisition and valuation of authoritative knowledges and their sources based on individual-level characteristics.

## 2 Methods

### 2.1 Research design and study context

This study included data from pregnant participants in the Stress, Pregnancy and Health (SPAH) observational study (R01MD011749). Recruitment eligibility criteria included being 18+ years of age, gestational age (GA) of <25 weeks, singleton pregnancy, English speaking, and planning to deliver within a single hospital system in Chicagoland, Illinois, USA. SPAH participants completed 2 study visits during pregnancy that included surveys, biometry, and venous blood collection, and consented to placental collection and medical record abstraction after delivery.

### 2.2 Data collection: understanding authoritative knowledge acquisition and valuation

#### 2.2.1 Interview recruitment

Participants for this sub-study were recruited via text message or phone call upon completion of their second prenatal study visit. Interview participants were recruited to discuss their knowledge of breastfeeding, regardless of their feeding intention. Interview participants were also recruited to ensure similar numbers across racial and ethnic identities, socioeconomic groups, and the inclusion of both primiparous and multiparous individuals. Categories for race included Black, white, Asian, other, and more than one race, and the only relevant ethnic category was Hispanic (Table 1). Participants could identify as more than one racial/ethnic group (e.g., Hispanic, other; Table 2). For semi-structured interviews, 25 participants were needed to reach saturation (34–36).

**Table 1:** Participant socio-demographics for qualitative interviews (*n* = 25).

Participant demographics	Mean (SD)/*N* (%)
Age	
Years	34.08 (4.2)
Race	
White	9 (36%)
Black/African American	3 (12%)
Asian	6 (24%)
Other	2 (8%)
American Indian	1 (4%)
More than 1 race	1 (4%)
No race reported	2 (8%)
Ethnicity	
Hispanic/Latine	5 (20%)
Partnered	23 (92%)
Parity	
First pregnancy	8 (32%)
First child	10 (40%)
2nd child	12 (48%)
3rd child	3 (12%)
Profession	
Education	8 (32%)
Healthcare	4 (16%)
Finance	2 (8%)
Administrative	2 (8%)
Stay at home/homemaker	2 (8%)
Law	1 (4%)
Graduate student	1 (4%)
Military	1 (4%)
Unemployed	1 (4%)

**Table 2:** Participant IDs and relevant characteristics for qualitative interviews (*n* = 25).

Participant ID	Age	Race, ethnicity	Parity
AK001	38	White	3rd Child
AK002	25	White	2nd Child
AK003	28	White	1st Child
AK004	39	White	2nd Child
AK005	35	Hispanic	1st Pregnancy
AK006	31	Hispanic	3rd Child
AK007	36	White	2nd Child
AK008	40	White	1st Pregnancy
AK009	36	White	1st Child
AK010	46	White	1st Pregnancy
AK011	43	Black	1st Pregnancy
AK012	36	Black	2nd Child
AK013	33	White	1st Pregnancy
AK014	34	Asian	2nd Child
AK015	34	Black	1st Pregnancy
AK016	32	Asian	2nd Child
AK017	29	Other, Hispanic	1st Pregnancy
AK018	29	Asian	2nd Child
AK019	26	Asian	2nd Child
AK020	45	Asian	2nd Child
AK021	29	American Indian, Hispanic	1st Pregnancy
AK022	34	Asian	2nd Child
AK023	23	Other, Hispanic	2nd Child
AK024	39	More than 1 race	3rd Child
AK025	32	Other	2nd Child

#### 2.2.2 Sub-study activities

To characterize participant acquisition and valuation of information sources about breastfeeding, participants completed a 30-to-60-minute phone-based semi-structured interview in their third trimester of pregnancy between November 2020 and March 2021. Interviews were audio recorded with permission, and participants received a $25 e-gift card upon completion of the interview. Participants were asked open-ended questions about: infant feeding plans and how they made those decisions; how they would define breastfeeding; what sources of information they used to prepare for breastfeeding; and how much they valued those sources by ranking them from most to least useful.

#### 2.2.3 Analysis

Audio recordings of interviews were uploaded and transcribed (MB) using Otter.ai, Inc. (2022, Mountain View, California). Atlas.ti Mac (Version 9.1.3) was used for coding. *A priori* themes from interview topics listed in the above section were used to inform the iterative development of codes by the authors (Table 3). Two coders collaboratively categorized the sources of information participants used to acquire information, and participants' valuation of those information sources (37). These sources of information led to the designation of three domains of authoritative knowledge.

**Table 3:** Relevant *a priori* themes and subsequent codes used for development of authoritative knowledge domains.

Themes	Codes
Infant feeding	• Breastfeeding; breastfeeding duration; exclusive breastfeeding; bottle feeding; mixed feeding; exclusive pumping; donor milk feeding; formula feeding; reason for choosing feeding mode.
Information source	• “I don't know”; “Expert”; books; cultural practices; doctor/healthcare provider; education/training; occupation; family members; friends; “from the hospital”; insurance company; “Googling”/internet searches; lactation providers; lay birth worker (e.g., doula); News media; other; popular media; pregnancy apps/websites; prenatal classes; religion/religious practices; research-based topical websites/blogs; social media/online communities; videos.
Quality of information	• Trust (least, less, more, most); reason for trust; reason for mistrust; “I trust”; “I don't trust”; value (least, less, more, most); “I value”; “I don't value”.
Type of information	• Alternative; anecdotal/experiential; avoided; conflicting; generalized; helpful; medical/expertise; multiple sources; no one talked about; previous knowledge/experience; unhelpful; none; other.
Sociodemographic information	• Index pregnancy; previous pregnancy; time since last delivery; number of children; parity; occupation status; maternal age; insurance status; single parent.

#### 2.2.4 Ethical approval

IRB approval was obtained from Northwestern University's IRB for SPAH (STU00206269), Evanston Hospital's IRB for SPAH (EH17-006) and a reliance agreement (STU00215233).

## 3 Results

### 3.1 Sociodemographic information

Twenty-five individuals participated in semi-structured qualitative phone interviews. The average age of participants was 34 years (range: 25–46 years, standard deviation: 4.2), 92% were partnered, and 60% had given birth before and raised a child (Table 1). Twenty-four (96%) of the participants planned to feed their newborn human milk and 23 (92%) anticipated attempting to feed from the breast, as 1 participant planned to exclusively feed donor milk, and 1 participant planned not to breastfeed. Lastly, 5 participants identified themselves as having medical training (e.g., midwife, doctor, nurse), with 4 working in healthcare at the time of their interview, and 1 participant having midwifery training from outside of the US.

### 3.2 The three domains of authoritative knowledge

**Figure 1:**
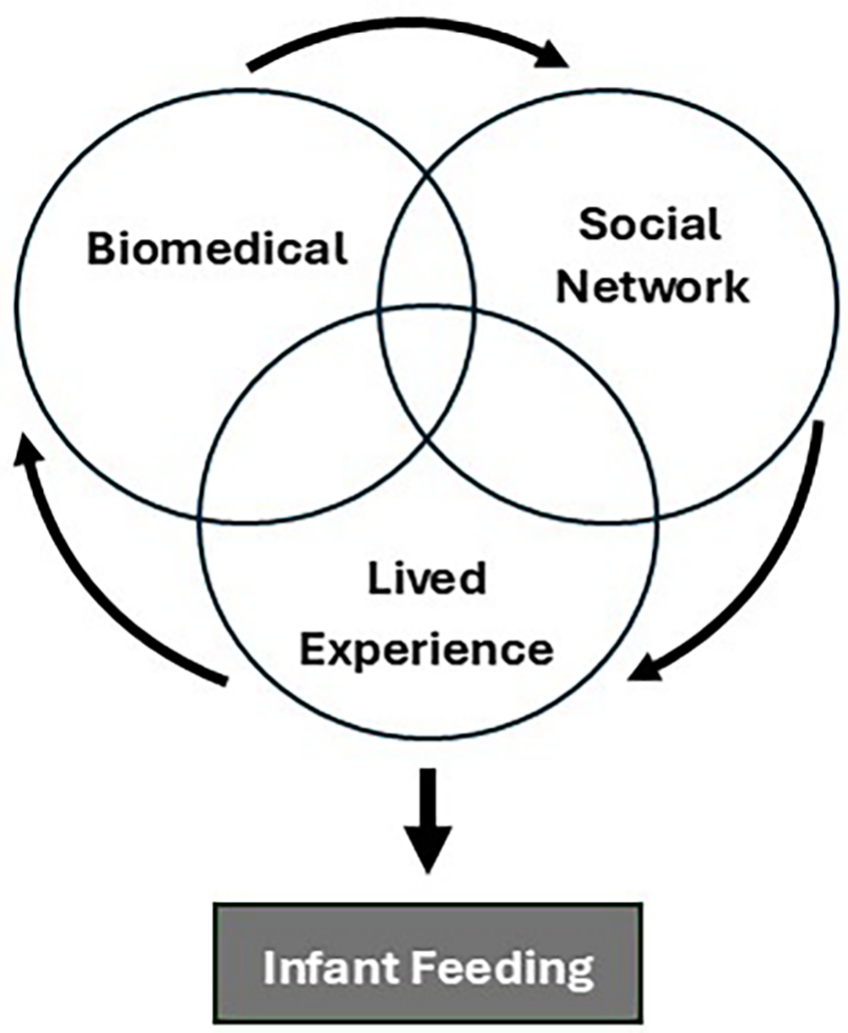
This conceptual framework shows how the three domains of authoritative knowledge defined by authors interacted and overlapped to influence participant preparation for infant feeding.

We identified three domains of authoritative knowledge based on code counts of the most used sources of information across the 25 interviews (Figure 1, Tables 3, 4). All 25 (100%) participants used both biomedical knowledge and social network knowledge in preparation for infant feeding. All 15 (60%) participants who previously had children also used lived experience knowledge. Inclusion of Tables 4, 5 allow for more robust reporting of participant responses.

**Table 4:** Illustrative quotes about breastfeeding presented by the three domains of authoritative knowledge.

Domain 1. Biomedical
“At least for my health, my and my child’s health, I want to know where the science is.”—AK017
“I try to look for something that’s more of like a medical lens, than like an individual or a kind of more naturalist approach.”—AK007
“I feel like there's just so much more reliable knowledge”—AK016
“But the NICU nurses really helped me realize what a latch was and what a latch wasn't. They’re like, because [your] son's tongue-tied. And so, I know you can breastfeed when your kid's tongue-tied.”—AK002
“But during our appointments, I feel like they’re so quick that I’m trying to like pepper her with questions […] sometimes I don’t feel like brushed off, but I feel like she’s like, ‘Oh, no, this is fine. You’re having a very routine pregnancy’, and okay, that might be the case right now [chuckles] but I need to prepare for these other scenarios, too.”—AK003
Domain 2. Social Network
“No, no. Not books and classes. I inquired like my seniors, my families, my mom, like about their experience and stuff.”—AK018
“I learned about the cons about [breastfeeding], like a woman she was talking about well you know, even when you’re not breastfeeding and you gotta pump the milk out and it’s gonna be leaking out of you and your nipples gonna be [in] pain and you may get an infection and it’s gonna be sore and it just don’t stop”—AK015
“I feel like she gives me more accurate info. I feel like [hearing] from an actual person that’s had the experiences and you know knowledge of it, I’ll take that much better than you know an article.”—AK006
“I think talking to, you know, friends and family who’ve recently had children, changed my thoughts on being prepared for whatever happened.”—AK011
Domain 3. Lived Experiences with older children	
“I know than I can because I breastfed with my first. I can make supply based on how they drink.”—AK019
“I’m hoping it’s an easier go this time, but I’m trying to keep my expectations low. I really don’t know, but I do feel more equipped, having been through it once, and knowing that I can, that there are multiple modes [of infant feeding].”—AK004
“so I plan on breastfeeding, and I feel like I did learn enough from my first experience where I know the areas where I needed to improve the most, which I think the biggest thing was like just not pumping enough […] I think, if it happens, great, then we’ll continue on, and I’ll try to do it a lot longer this time than I did last time.”—AK016
“I’m hoping he’ll breastfeed and he’ll be different from his sister […] I don't produce a lot of milk, if he does breastfeed he’ll still have to be supplemented by formula.”—AK020

**Table 5:** Illustrative quotes demonstrating the intersections of the three domains of authoritative knowledge.

Illustrative quotes	Domains
“My own experiences, I realized that the physical and mental health of the mother plays into [breastfeeding] … just knowing I don't have to do exactly what my friends are doing [for example] I have friends who’ve breastfed for 16 months and maybe in the past I would’ve been like ‘oh, maybe I should do that’ but [now] I’m like ‘that's great for you—I’m not going to do that’.”—AK024	Lived experience; social network
*Reflecting on previous experience of preparing to breastfeed*: “So I have information, I know I can produce milk. Hopefully. I knew my nipples were getting engorged and [I was] making colostrum. But I wanted to experience, what it feels like, so I would ask a friend.”—AK019	Biomedical; social network; lived experience
“I told you I come from India so I have a really big family. I knew that breastfeeding is a thing, that your supply is regulated by their demand. I had a lot of social and cultural exposure that added to the knowledge that humans are mammals and mammals produce milk.”—AK019	Social network; biomedical
“And just spreading out how I learned with the internet, friends, family, and my doctors made sense to me instead of just one source.”—AK013	Social network; biomedical
“Whereas [the] Facebook group will [offer] so many different things you can do or try. But they wouldn’t condone that in the webinar, they don’t really mention or say that those things would work, it’s mainly just continue to latch more frequently to get your milk to flow more.”—AK012	Social network; biomedical
“Yeah, I think before I took the class, a few of my friends will just breastfeed and pump non-stop and have all kinds of supplies. And after going through the class, the lactation consultant was like, ‘No [laughs] if you’re like going to be home that’s not how it goes’. So, I had a different picture of what breastfeeding and pumping was like, [the class] kinda changed my mind a little, or my thoughts around it.”—AK005	Biomedical; social network
“I follow like OB-GYN influencers,”—AK013	Biomedical; social network
“And they all have babies, although they are not educated as the OB, they have the personal experience so they can share that side information with me.”—AK014	Biomedical; social network
“I think talking to, you know, friends and family who’ve recently had children, changed my thoughts on being prepared for whatever happened. So, some people had issues breastfeeding, some people experienced pain, some people had to supplement with formula. Like a friend said that they introduced her son to the bottle in the hospital, and then she was never able to like actually properly nurse him. So it’s just given me a wider range of understanding of all the things that could happen.”—AK011	Social network; biomedical
“I feel like she [sister-in-law] gives me more accurate info. I tell her like, well, I researched this, and this article says this, and this article says that, then I’m like, what is your take on this? I feel like [hearing] from an actual person that’s had the experiences and you know knowledge of it, I’ll take that much better than you know an article.”—AK006	Biomedical; social network
“[scientific articles] are very difficult to read through, and then I supplement them with abstracts of other ones, and then the personal opinions of people online to see how those go together.”—AK013	Biomedical; social network

### 3.3 Authoritative knowledge domain (1) biomedical

Biomedical information was used by every participant (*n* = 25) and provided by sources including prenatal care professionals (e.g., obstetricians, midwives), the healthcare system (e.g., insurance, hospital-based support services), public health services for families with lower socioeconomic status (e.g., Special Supplemental Nutrition Program for Women, Infants and Children or WIC), lactation consultants, and other biomedical and public health experts. This domain also included resources maintained by healthcare institutions or backed by scientific research (e.g., websites and journal articles), as well as participants' own personal education and career training (e.g., medical school, midwifery training).

All interview participants recognized that human milk was the healthiest first food for infants. Only one participant planned not to breastfeed at all, partially due to concerns over returning to work. Some participants viewed lactation as a scientific process, and thus they sought out and prioritized scientific sources to make their infant feeding choices. For example, a participant (AK013) described how their husband who works in the biomedical field would find them articles from “first tier journals” to learn about breastfeeding (Table 4). Participants underscored the importance of prioritizing science in making infant feeding decisions.

Participants consistently prioritized biomedical sources as the most valuable and trustworthy source of information regarding breastfeeding recommendations or troubleshooting issues with lactation.

“[The first source of information I would turn to] would be my provider, so that I can get more accurate, secure information, I want to say like more reliable […] because I’m not the only person they took care of.”—AK021

Though it was the most valued, biomedical knowledge was sometimes the hardest to access because time with the healthcare provider was limited to a handful of brief prenatal care appointments.

“I just feel like maybe stuff is like rushed through and I don’t get like, all the information or I feel like I’m being rushed and I don’t get to ask all my questions. But I don’t think it’s done intentionally.”—AK005

### 3.4 Authoritative knowledge domain (2) social network

Social network information was also used by every participant (*n* = 25) and came from individuals in participants' social networks (e.g., friends, family), and people on the internet who may or may not be known personally to the respondent. This domain also included online and media-based information including social media platforms (e.g., Instagram, TikTok, Facebook), and pregnancy apps (e.g., Ovia, BabyCenter, Tinyhood), and pregnancy websites with chat or discussion functions (e.g., What to Expect). Pregnancy websites and apps were included in this domain because participants discussed the utility and value of the social aspects more than the actual information provided about topics such as infant feeding.

While many participants preferred information from those they knew personally, finding their friends, family, and other direct connections to be more accessible and relatable.

“[My friends] all have babies, although they are not educated as the OB, they have the personal experience so they can share that side information with me.”—AK014

Others preferred receiving information from those they did not know personally, as long as they could connect with an individual's story (Table 4). The only participant (AK015) who decided against breastfeeding before delivery explained this decision was made in part by listening to strangers' experiences of pain during lactation via the social media platform TikTok (Table 4). Participants felt internet-based resources were valuable because they provided broad access to birthing people's experiences of parenthood, giving them a better sense of what it could be or feel like to experience breastfeeding, as well as finding tools that may functionally improve breastfeeding, like recommendations for breast pumps or pillows. For example, the What to Expect app provides forums where birthing people can post questions and answers to questions, demonstrating how others' experiences can be shared across large groups of people who do not actually know one another.

### 3.5 Authoritative knowledge domain (3) lived experiences with older children

More than half (*n* = 15) of participants set their expectations for their current pregnancy based on prior lived experience and they all used these experiences to inform preparation for their upcoming infant feeding journeys. Some participants had successfully breastfed a child in the past and believed that would lead to success in their upcoming postpartum period.

“I think having a good experience [with breastfeeding] the first time makes me feel like that will be something that I can do again.”—AK007

There were also participants who were unsuccessful with breastfeeding in the past but hoped to breastfeed successfully this time despite past experiences. Those with prior infant feeding experiences that did not align with their goals often accessed information more proactively to mitigate future issues. As one participant (AK012) described their first infant feeding experience, where a pediatrician recommended formula feeding because their baby was losing weight,

“I think I may have breastfed for maybe 3–4 weeks and then changed it to strictly formula with him. But I’ve been preparing myself more to breastfeed, and I plan to do it for a while.”—AK012

During this pregnancy, this participant joined Facebook groups for Black breastfeeding moms and watched breastfeeding webinars on YouTube to prepare for their upcoming infant feeding journey. There were, however, some participants who took a more relaxed attitude and did minimal additional research.

“I’m hoping it’s an easier go this time, but I’m trying to keep my expectations low. I really don’t know, but I do feel more equipped, having been through it once, and knowing that I can, that there are multiple modes [of infant feeding].”—AK004

### 3.6 Intersecting domains of knowledge

Pregnancy tracker apps and social media platforms sometimes served as a source of “evidence-based information” i.e., biomedical knowledge when participants sought out media content created by healthcare professionals (doctors, nurses, lactation consultants) on social media platforms or podcasts. For example, one participant accessed evidence-based information from birth workers accounts on Instagram (Table 5).

Conversely, several participants appreciated when their healthcare provider shared about their own pregnancy and breastfeeding experience, finding this information to be authoritative because of the purveyor and context (their doctor during a prenatal visit) as well as relatable.

“[My obstetrician has] been very good about answering my questions. And sometimes I was like, “oh, what do you recommend?” She’s like ‘I can’t actually recommend one thing or another as your doctor, but as a woman that has given birth, I can tell you that this is my experience.’ And that’s been helpful.”—AK013

## 4 Discussion

In this first application of authoritative knowledge within the context of infant feeding, we identified three domains of authoritative knowledge pertinent to breastfeeding: biomedical, social network, and lived experience. All 25 (100%) participants acquired knowledge from multiple biomedical and social network sources, and 15 (60%) relied on their own lived experience from previous children to prepare for breastfeeding their new baby.

Participants consistently cited biomedical authoritative knowledge as the most accurate and important. However, those without personal medical expertise described limited access to biomedical authorities during prenatal care due to the short duration of prenatal appointments and the challenge of completing prenatal education courses. The lack of access to evidence-based prenatal education in the US has been reported previously (38), with slightly more than half of all first-time parents completing any type of prenatal education (39). The high-throughput model of medical care in the US provides limited time for effective patient education, and medical training does not prioritize competence in the provision of comprehensive, patient-centered education (6, 40). Prenatal education is typically offered external to routine prenatal care appointments, for a fee, and sometimes not even near the hospital or clinic (41, 42). A survey-based study of 59 pregnant individuals identified that transportation, childcare, and costs were routine barriers for individuals to receive education prior to delivery (38).

It is unsurprising, then, that participants turned to social network authoritative knowledge, including internet- and app-based sources, for information about breastfeeding. The advantages of social network authoritative knowledge about breastfeeding was its accessibility and, perhaps more importantly, its relatability. Participants had a strong desire to learn what breastfeeding was really going to be like from those who were currently experiencing it. Though it was important to hear the benefits of breastfeeding, participants wanted practical advice about preparing themselves for breastfeeding success, managing their supply, and navigating return to work. Friends, family, and even strangers on the internet were almost exclusively sources of this information, though as was mentioned above, at least one participant (AK013) appreciated hearing their physician share their personal experiences. These findings reflect why breastfeeding peer counseling (4) a strategy of providing breastfeeding support and guidance via a community health worker with shared, lived experience, remains a highly effective strategy for improving breastfeeding outcomes (43–45), particularly when racially/ethnically concordant (46, 47). Similarly, group prenatal care has been associated with improved breastfeeding outcomes, in part due to shared learning strategies (48).

Lived experience was an important source of authoritative knowledge for the participants (*n* = 15) who had older children, and the variability in those prior expeirences gave participants different expectations and approaches to breastfeeding preparation in their subsequent pregnancy. Those who had a prior negative experience were more likely to express concern about their ability to breastfeed their next child, though some shared their motivation to overcome the barriers from last time. This finding highlights the potential importance of prenatal breastfeeding education for all parents, and not just first time parents. At least one prior article found that multiparous individuals who had a negative prior breastfeeding experience were more likely to terminate breastfeeding earlier in a subsequent pregnancy (49), which could result from less motivation to try or a sense of hopelessness that they could have a better experience with the next baby (50). Peer and group models of care could bring together primiparous and multiparous individuals, allowing for the intersection of this domain with social network authoritative knowledge. We acknowledge lactation challenges are often multi-factorial and that knowledge is just one of the solutions needed to support parents' infant feeding goals.

Finally, we identified that social media was a space where social and biomedical authoritative knowledges often overlapped. Many participants sought evidence-based biomedical information about breastfeeding on apps, social media, and websites. While the use of pregnancy apps and websites is advocated for by professional organizations such as The American College of Obstetricians and Gynecologists, content and quality across platforms varies significantly (51–53), and these may be an important area of innovation to improve access to reliable and helpful breastfeeding information.

### 4.1 Limitations

There are some limitations to note. Interviews took place during the Covid-19 pandemic, during which healthcare models for prenatal and intrapartum care significantly reduced direct contact, limiting social support during birth and postpartum, and restricting access to sources of information like doulas and in-person prenatal classes (54, 55). The participants included in this study were recruited from a single hospital-system in the Chicagoland area and were already participating in a research study, so may not be generalizable to other patient populations within or outside of the US. Additionally, 5 of the 25 participants had healthcare backgrounds which may also affect generalizability of the results. Participants were not explicitly asked about their cultural and ethnic backgrounds; therefore, future work should explicitly focus on the variability in use of information sources by socio-demographic characteristics.

## 5 Conclusion

We identified three domains of authoritative knowledge—biomedical, social network, and lived experience—that interacted to shape participants' expectations for breastfeeding during pregnancy. Our results suggest that biomedical information sources were valued as the most authoritative (56, 57), but that social network authoritative knowledge was more accessible and informative about the actual experience of breastfeeding. This study's findings can guide future efforts to increase relevance and accessibility of prenatal breastfeeding education via expansion of peer counseling and group prenatal care and education models, as well as think more creatively about how to communicate both evidence-based information and lived experiences to parents and families.

## Data Availability

The datasets presented in this article are not readily available because IRB approval was not received to share these data. Requests to access the datasets should be directed to butlerms@uic.edu.
